# Production of Uniformly Sized Gallium-Based Liquid Alloy Nanodroplets via Ultrasonic Method and Their Li-Ion Storage

**DOI:** 10.3390/ma14071759

**Published:** 2021-04-02

**Authors:** Chenghao Huang, Junjie Zong, Xiaodong Wang, Qingpin Cao, Dongxian Zhang, Jian-Zhong Jiang

**Affiliations:** 1 International Center for New-Structured Materials (ICNSM), Laboratory of New-Structured Materials, State Key Laboratory of Silicon Materials, School of Materials Science and Engineering, Zhejiang University, Hangzhou 310027, China; huangch@zju.edu.cn (C.H.); zongjjzju@163.com (J.Z.); wangxd@zju.edu.cn (X.W.); caoqp@zju.edu.cn (Q.C.); zhangdx@zju.edu.cn (D.Z.); 2 State Key Laboratory of Modern Optical Instrumentation, Zhejiang University, Hangzhou 310027, China

**Keywords:** Ga-based liquid alloys, nanodroplets, ultrasonic technique, surfactant, centrifugation, Li-ion battery

## Abstract

Gallium-based liquid alloys are attractive due to their unique properties, and they can potentially be applied in the field of flexible electronics as coolant materials for nuclear and liquid batteries, due to the high thermal conductivity and excellent fluid properties of liquid metals. However, it is still challenging to fabricate gallium-based liquid alloy nanodroplets with uniform and small size. Here, we performed a systematical study on the influence of various factors affecting the size of nanodroplets. Liquid metal nanodroplets with an average size of 74 nm and narrow size distribution were successfully fabricated. Li-ion half-cells were assembled with eutectic GaIn (eGaIn) nanodroplets as anode active materials, which showed higher specific capacity than the bulk eGaIn alloy under the same testing conditions.

## 1. Introduction

The concept of liquid alloys has a long history [1]. Generally speaking, in the periodic table of elements, post-transition elements, zinc group elements and their alloys, and Group IA elements and their alloys belong to this kind of emerging materials, such as Ga, GaIn, Hg, NaK, etc. They have both metallic and liquid properties, such as fluidity, high conductivity, and high thermal conductivity. Compared with amalgam [2] and alkali metal elements, gallium-based alloys have attracted much attention due to their low toxicity, low volatility, and biocompatibility. At present, nanostructured gallium-based liquid alloys, e.g., nanodroplets, have many applications in the fields of reconfigurable electronics, soft robotics, and conformable medical devices [3,4,5]. For example, it has been proved that adriamycin (DOX)-treated ultrasonic gallium-based alloy nanodroplets can be used for tumor-targeted therapy and enhance the chemotherapy efficacy of tumor-bearing mice [6]. In addition to biomedical applications, nanodroplets of gallium and its alloys are often used in sensor devices to increase the surface to volume ratio for significant improvement of the sensitivity [7].

Although gallium-based alloy nanodroplets have many applications, the issue of how to prepare these nanodroplets or even high-quality nanodroplets (uniform narrow size distribution and small average size below 100 nm) is still challenging. Microfluidics produced uniform liquid alloy microdroplets [8,9,10,11], but with limited effects on the size of liquid alloy droplets [12]. Physical vapor deposition was also used to prepare liquid alloy nanodroplets on various substrates. The most common strategy is to disperse bulk liquid alloys to micro or nano scale by an ultrasonic technique in an appropriate solvent with surfactants. Hohman et al. found that ultrasound treatment could effectively emulsify liquid metal, and assist the formation of liquid metal nanodroplets through sulfhydryl self-assembly [13]. Finkenauer et al. studied the effects of solvents and surfactants on the formation and stability of gallium-based alloy nanodroplets in an ultrasonic method, and found that ethanol and octadecanethiol were better choices [14]. However, the nanodroplets obtained by these methods are still large in size, and highly uneven with a wide size distribution.

In this work, the effects of various factors on the preparation of gallium-based alloy nanodroplets by an ultrasonic method will be systematically investigated, especially ultrasonic amplitude, time, and surfactant concentration, among other factors. In addition, eutectic GaIn (eGaIn) nanodroplets can be applied as active electrode material for Li-ion batteries owing to their high theoretical capacity and self-healing properties as compared with bulk eGaIn liquid. Thus, a half-cell Li-ion battery using eGaIn nanodroplets prepared here was assembled, which shows average discharge specific capacity of 582.6 and 379.6 mAh g^−1^ at a current density of 0.1 and 3.0 A g^−1^, respectively, which is much better than bulk eGaIn.

## 2. Materials and Methods

### 2.1. Synthesis of Eutectic GaIn Nanodroplets

**Figure 1:**
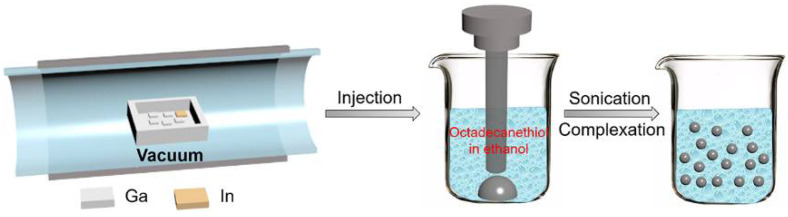
Schematic illustration of the fabrication process of eutectic GaIn (eGaIn) nanodroplets.

For the preparation of a eutectic GaIn alloy (eGaIn, 85.8 at.% Ga and 14.2 at.% In), pure gallium and indium with purity higher than 99.99 at.% were used. The mixtures of pure gallium and indium were sealed in quartz tubes under a vacuum of ~5 × 10^−4^ Pa and heated at 250 °C for a few hours until complete melting in a thermostatic oil bath. After melting, the alloys were cooled to room temperature and transferred into sealed vials using a plastic transfer pipette for further use. The second step is to prepare eGaIn nanodroplets. In this process, 0.2 g of eGaIn alloy and 20 mL surfactant solution (octadecanethiol dissolved into ethanol) are dropped into a 25 mL beaker. Nitrogen gas was used to remove oxygen for 5 min to improve combined efficiency between ligand and liquid alloy. Then, eGaIn alloy was synthesized into nanoparticles by performing ultrasonication with the Sonics Vibra-Cell Ultrasonic Processor VCX 750 (Sonics&Materials Inc, Newton, MA, USA) for different times (1, 2, 4, 6, and 12 h). Ultrasonication processor was operated at 750 W, 20 kHz, and different amplitudes (30%, 40%, and 50% of the maximum). In the ultrasonic process, an ice bath was used to mitigate the temperature fluctuation. The final solution was centrifuged to remove the surfactant and dropped onto a silicon wafer. The synthesis process of eGaIn nanodroplets by sonicating the bulk eGaIn alloy precursor is schematically illustrated in Figure 1.

### 2.2. Material Characterization

The nanodroplets were dropped onto a silicon wafer for structural characterization. After the ethanol volatilized, scanning electron microscopy (SEM, Zeiss Supra 55, Zeiss, Heidenheim an der Brenz, Germany) equipped with an energy dispersive X-ray spectrometer (EDS) was used to characterize the morphology and composition, respectively, and Nanomeasure (Nano Measurer 1.2, Nanomeasurer is developed by Department of Chemitry, Fudan University, https://nano-measurer.software.informer.com/ (accessed on 1 April 2021), Shanghai, China) software was used to calculate the size distribution of nanodroplets. It was found that all droplets prepared here have the same composition as the bulk liquid alloy.

### 2.3. Electrochemical Measurements

A slurry was made by mixing the nanodroplets, super P carbon black, and polyvinylidene fluoride (PVDF) binder in N-Methyl pyrrolidone (NMP) solution at a mass ratio of 5:4:1 by stirring for 24 h. The slurry was dropped onto Cu foil and dried in a vacuum oven at 60 °C for 12 h. The mass loading of the active materials (eGaIn nanodroplets)on the electrode is about 0.7~1.0 mg. The 2025-type coin was assembled with eGaIn nanoparticles electrode and Li metal in a glove box filled with Ar gas (H_2_O < 1.0 ppm, O_2_ < 1.0 ppm). The electrolyte used for the Li-ion test was 1 M LiPF_6_ dissolved in ethylene carbonate/ethylmethyl carbonate/dimethyl carbonate (EC:EMC:DMC = 1:1:1 by volume). Galvanostatic charge–discharge tests were performed by LANDdt software (CT2001A, LANHE, Wuhan, China, voltage range: 0.005–2 V vs. Li^+^/Li). For comparison, bulk eGaIn alloy was also applied for Li-ion storage tests under the same conditions.

## 3. Results and Discussion

**Figure 2:**
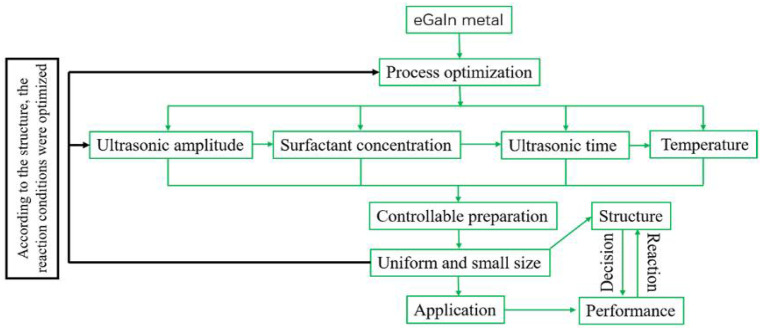
Schematic plot for the synthesis strategy of eGaIn nanodroplets.

Bulk eGaIn alloy precursors were synthesized by a simple annealing method in a vacuum. In order to obtain droplets with smaller size and narrower size distribution, the effects of ultrasonic amplitude, surfactant concentration, total ultrasonic time, single ultrasonic time, and centrifugal speed were studied as shown in Figure 2.

### 3.1. Ultrasonic Amplitude

**Figure 3:**
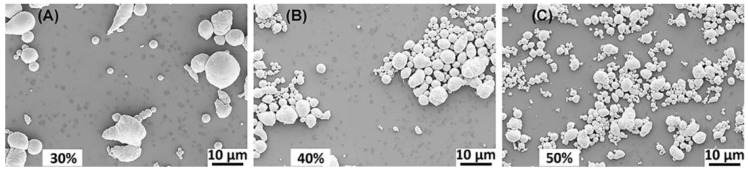
Morphology images of eGaIn nanodroplets under different ultrasonic amplitudes: (**A**) 30% (**B**) 40%, and (**C**) 50%.

Because ultrasonic amplitude was an important parameter, which provides enough kinetic energy to break the bulk eGaIn liquid alloy to form sufficiently small droplets, we first studied the factor of ultrasonic amplitude. Experimental conditions: different percentages 30%, 40%, and 50% of the maximum amplitude, the same surfactant concentration of 1 mmol/L, the same ultrasonic time of 1 h, and ice bath to keep the temperature at about 35 °C. The SEM images for the morphology of droplets prepared by 30%, 40%, and 50% amplitudes are shown in Figure 3. It is clear that the sample using 30% amplitude has many larger droplets up to 10 μm, and 40% amplitude further reduces the largest size. Using 50% amplitude, smaller droplets below 1 μm are formed. Thus, 50% amplitude is used for further tests.

### 3.2. Surfactant Concentration

**Figure 4:**
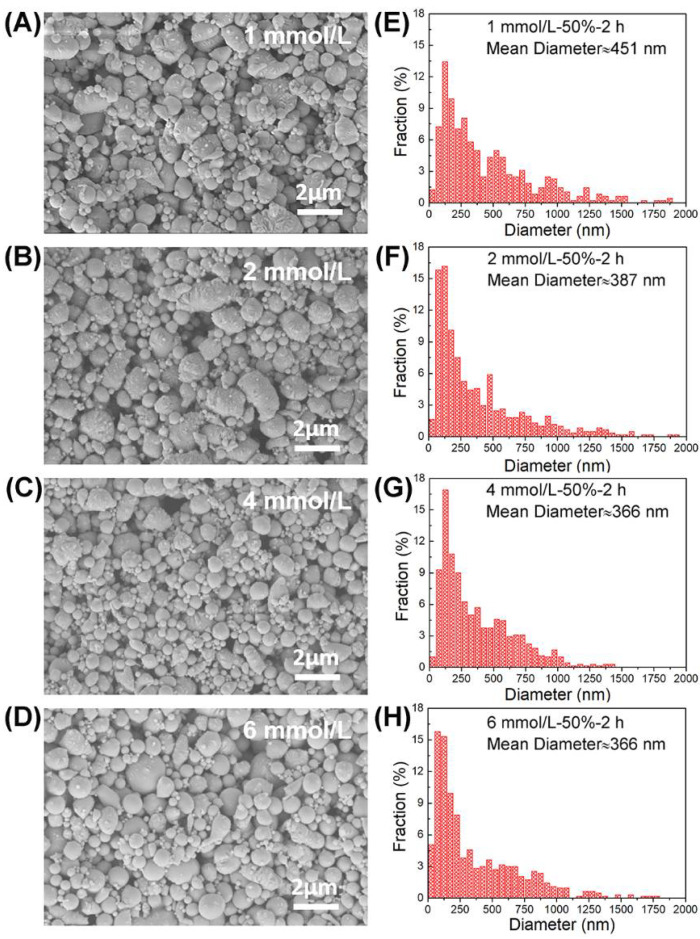
Morphology images and size distributions of eGaIn nanodroplets under different concentrations of surfactant: (**A**,**E**) 1 mmol/L, (**B**,**F**) 2 mmol/L, (**C**,**G**) 4 mmol/L, and (**D**,**H**) 6 mmol/L.

In the process of preparation, the role of the surfactant is to adsorb and stabilize droplets of eGaIn alloy, and hinder reunion between droplets. Experimental conditions: different concentrations of surfactant (1, 2, 4, and 6 mmol/L), the same ultrasonic amplitude 50% and the same ultrasonic time 2 h, and ice bath was used to keep the temperature at about 35 °C. Figure 4A–D show the morphology of nanostructured droplets as measured by SEM, while the droplet size distributions are plotted in Figure 4E–H. As shown in Figure 4A,B and the corresponding statistical in Figure 4E,F, respectively, more droplets with smaller size (below 200 nm) were observed for the concentration of 2 mmol/L, with an average size of 387 nm, as compared to the case for the concentration of 1 mmol/L, with an average size of 451 nm. When the surfactant concentration increases to 4 mmol/L and 6 mmol/L with the same average size of 366 nm, the amount of larger droplets with size larger than about 1000 nm decreases. During the ultrasound process, the bulk liquid alloy is broken and surfactant as a ligand is absorbed on the bare surface to retard the reunion of droplets. The higher the surfactant concentration, the higher the chance for ligand-covered droplets be smaller. It seems that a critical concentration value of about 2–4 mmol/L is preferred for the preparation of nanodroplets.

### 3.3. Ultrasonic Time

**Figure 5:**
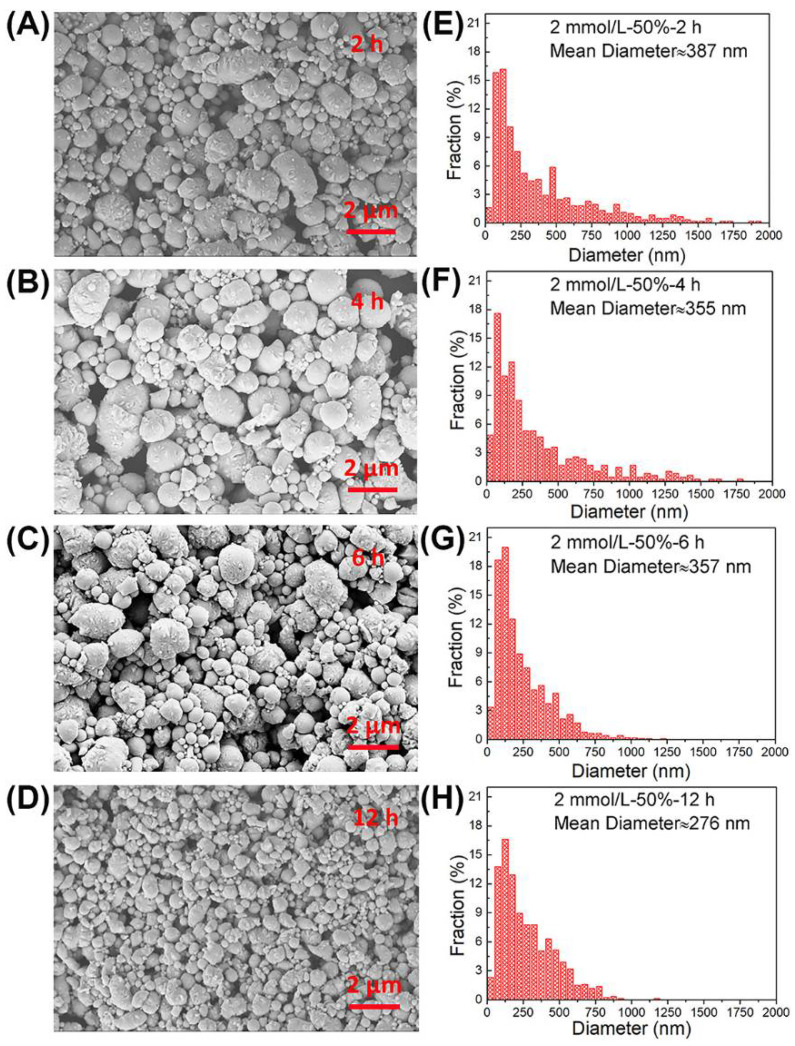
Morphology images and size distributions of eGaIn nanodroplets under different ultrasonic times: (**A**,**E**) 2 h (**B**,**F**) 4 h (**C**,**G**) 6 h and (**D**,**H**) 12 h.

Experimental conditions: under the same surfactant concentration of 2 mmol/L and the same ultrasonic amplitude of 50%, the same ice bath keeps temperature at about 35 °C during the ultrasonic process, different ultrasonic times of 2, 4, 6 and 12 h were selected. SEM morphology characterization and droplets statistics results are shown in Figure 5A–H. It is found that with increasing the ultrasonic times from 2 to 12 h, the average size of droplets is clearly reduced from 387 nm to 276 nm, respectively. For the ultrasonic time of 12 h, as shown in Figure 5D,H, the overall morphology of droplets is relatively uniform with almost none above 1 μm. It can be concluded that, in the whole ultrasonic process, before the liquid alloy reaches the equilibrium of crushing and re bonding, the longer the ultrasonic time, the more the crushing process, and the smaller the droplet size. Therefore, prolonging the time is a favourable factor in the ultrasonic process to obtain small size droplets.

### 3.4. Temperature in Solution

**Figure 6:**
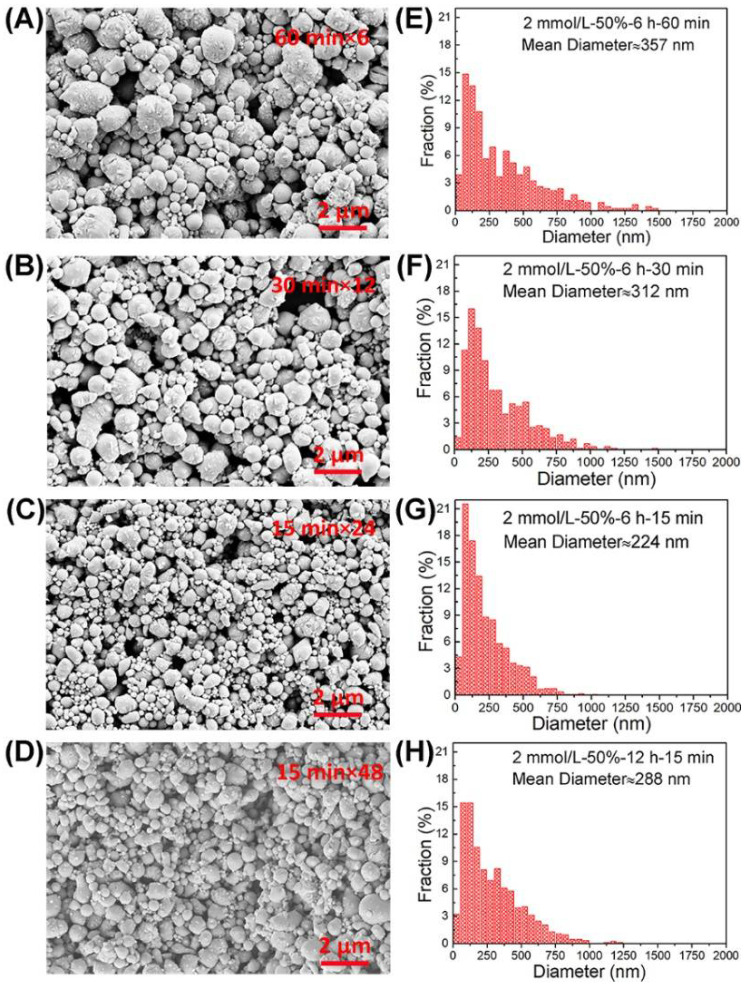
Morphology images and size distributions of eGaIn nanodroplets under different single ultrasonic durations: (**A**,**E**) 6 repeats of 60 min “on”, (**B**,**F**) 12 repeats of 30 min “on”, (**C**,**G**) 24 repeats of 15 min “on”, and (**D**,**H**) 48 repeats of 15 min “on”.

It should be noted that during ultrasonic oscillation, the temperature of the solution increases, indicating the local temperature at liquid alloy could be higher. Thus, temperature variability should be examined. Experimental conditions: the same surfactant concentration 2 mmol/L, ultrasonic amplitude 50%, and the total ultrasonic time 6 h; the total ultrasonic time is achieved via three different multiplying ultrasonic “on-off” modes, i.e., (1) 24 repeats of 15 min “on” and 5 min “off”, (2) 12 repeats of 30 min “on” and 5 min “off”, and (3) 6 repeats of 60 min “on” and 5 min “off”. Ice bath is always applied. SEM morphology characterization and droplet size distributions are plotted in Figure 6. From Figure 6A–C, large-sized droplets of about 1.5 μm are observed for the single ultrasonic duration of 60 min. When the single ultrasonic duration is 30 min, the large-sized droplet decreases to about 1 μm. When the single ultrasonic duration is further shortened to 15 min, the overall morphology becomes uniform and the fraction of small droplets largely increases. The same results are also reflected in the size distributions of droplets in Figure 6E–G with the average droplet size of 357 nm for 60 min, 312 nm for 30 min, and 224 nm for 15 min. Under the condition of constant total ultrasonic time, the shorter the single ultrasonic duration, the higher the proportion of small droplets, the lower the fraction of large droplets, and the smaller the overall droplet size. It can be understood that if the time is too long, the ultrasonic oscillation will increase the temperature of the solution. However, a short single ultrasonic duration means relatively lower temperature of the solution, which can reduce the probability of reunion events between droplets.

In addition, we also compared the total ultrasonic time of 6 h with 12 h under the same single ultrasonic duration (15 min), as shown in Figure 6C,D,G,H. It is found that when the ultrasonic time increases from 6 to 12 h, the average droplet size does not further decrease, but rather increases from 228 to 288 nm. These results indicate that it is important to ensure relatively lower temperature of the solution, and the single ultrasonic duration should not be too long for the preparation of eGaIn nanodroplets by the ultrasonic technique.

### 3.5. Centrifugal Process

**Figure 7:**
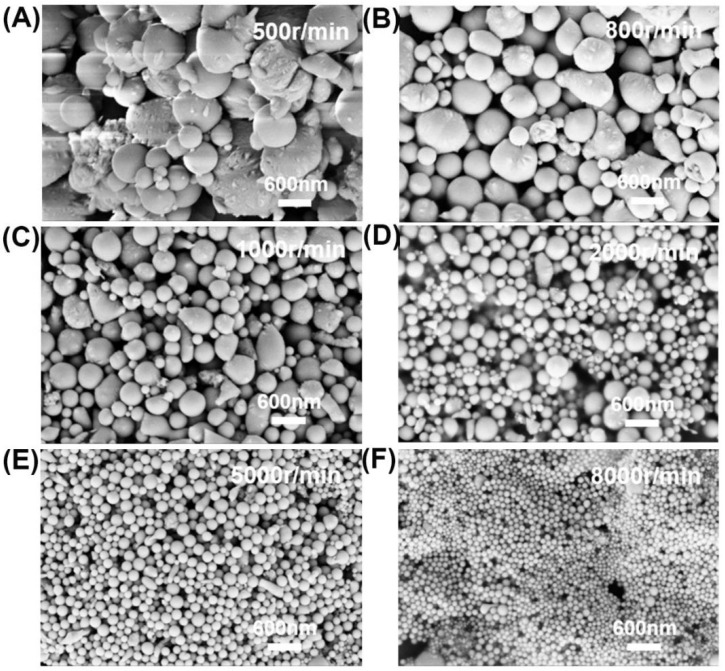
Morphology images of eGaIn nanodroplets under different centrifugal speeds: (**A**) 500 r/min, (**B**) 800 r/min, (**C**) 1000 r/min, (**D**) 2000 r/min, (**E**) 5000 r/min, and (**F**) 8000 r/min.

**Figure 8:**
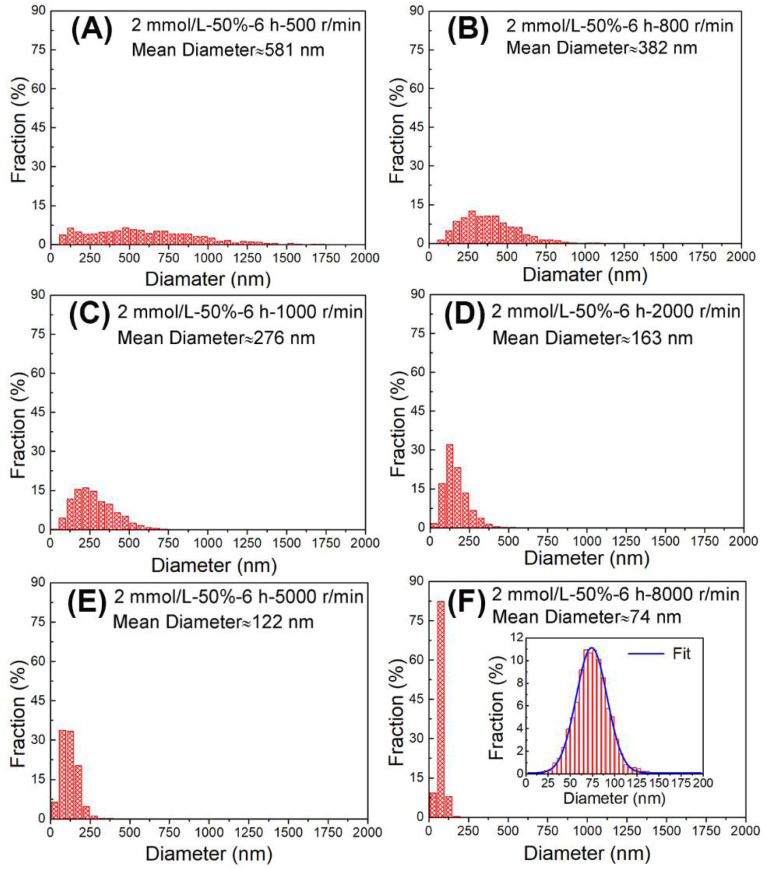
Size distributions of eGaIn nanodroplets under different centrifugal speeds: (**A**) 500 r/min, (**B**) 800 r/min, (**C**) 1000 r/min, (**D**) 2000 r/min, (**E**) 5000 r/min, and (**F**) 8000 r/min.

After all the above-mentioned efforts, droplet size distribution is still wide. To obtain high-quality liquid metal nanodroplets (i.e., uniform with smaller size distribution and smaller average size), we also tested various centrifugation speeds to separate the original solution. The experimental conditions were as follows: the concentration of surfactant was 2 mmol/L, the ultrasonic time was 6 h, and the ultrasonic amplitude was 50%. The different centrifugal speeds used were 500 r/min, 800 r/min, 1000 r/min, 2000 r/min, 5000 r/min, and 8000 r/min to centrifugate the original solution step by step, in which the upper solution of centrifugation was used for the next step. SEM morphology characterization and droplet size statistics are shown in Figure 7 and Figure 8, respectively. It is clear that the large size droplets are screened at low speed. When the centrifugal speed is 5000 r/min, the size distribution becomes uniform with an average size of about 122 nm, and for 8000 r/min, the size distribution gets even smaller with an average size of 74 nm. It is obvious that centrifugation is beneficial to separate nanodroplets with different sizes. However, due to nanodroplets with different sizes having different mass fractions, the yield is low when the centrifugation speed is low because the fraction of larger particles in the sample is smaller. The yield is large for the size of about 300–500 nm. Above 2000 r/min, the higher the centrifugation speed, the lower the yield. Hence, smaller and narrower size distribution of liquid eGaIn alloy nanodroplets can be fabricated by an ultrasonic technique using optimized parameters.

Based on the above analyses, the eGaIn nanodroplets exhibit smaller and narrower size distribution, which can be attributed to the following reasons: (1) High amplitude used can provide more energy to the liquid metal and a higher chance for the breaking of droplets. Consequently, smaller eGaIn nanoparticles are fabricated in Figure 3. (2) The thiolate surfactant can effectively prevent the oxidation of the nanodroplets and assist droplet cleavage to the nanoscale [13]. Although the droplet size will decrease with increased surfactant concentration, the higher concentration has less effect on the further reduction of the size [14]. Hence, a moderate concentration could be used. (3) Similar to the surfactant concentration, average diameters will decrease with increased sonication time. However, heat will accumulate as the ultrasound time increases, which will increase the nanodroplet size. Hence, it is difficult to reduce the droplet size further with prolonged ultrasonic time due to the balance between breaking and merging of droplets [15,16]. (4) Due to particle size distribution in the ultrasonic treated samples, nanodroplets with narrow size distribution can be obtained through high centrifugation speed, but the yield is low.

### 3.6. eGaIn Nanodroplets as Anode for Li-Ion Battery

**Figure 9:**
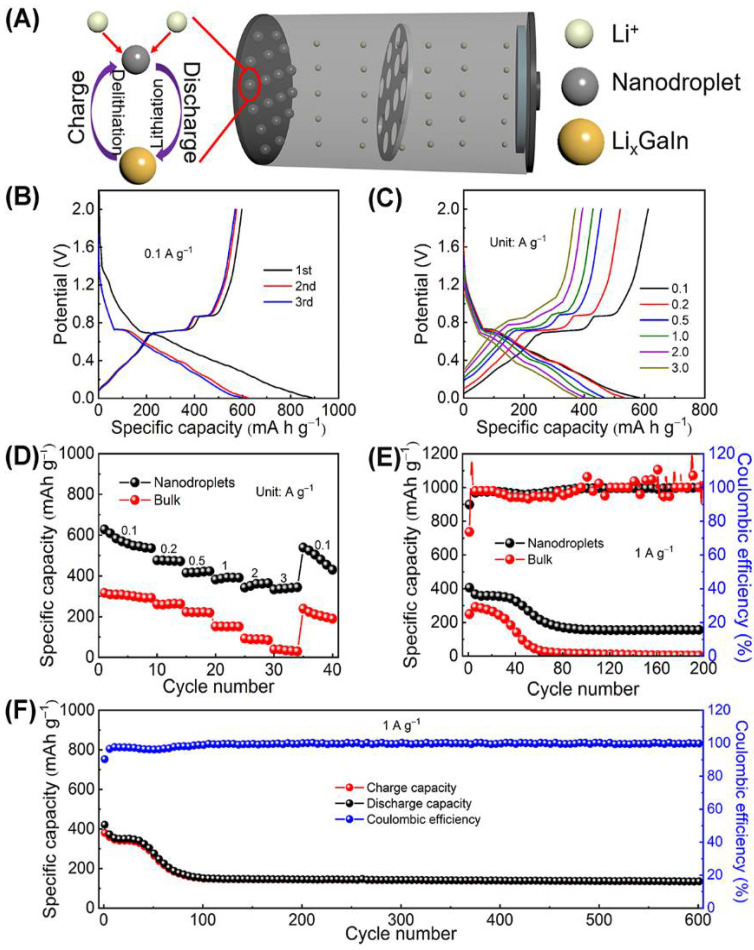
(**A**) Schematic illustration for the structure and the charge storage mechanism of Li-ion half-cell. (**B**) Charge–discharge curves at different current densities and (**C**) the first three charge–discharge curves at 0.1 A g^−1^ of eGaIn nanodroplets. (**D**) Cycling stability tests and (**E**) rate-capability tests of eGaIn nanodroplets and bulk eGaIn alloy. (**F**) Cycling performance of eGaIn nanodroplets.

Ga and In metals can form alloys with Li. When they are used as the anode active materials for a Li-ion battery, they could have higher theoretical capacity than commercial graphite anodes (372 mAh g^−1^). Hence, a Li-ion half-cell was assembled to evaluate the potential application of the eGaIn nanodroplets prepared here. Figure 9A shows the schematic illustration for the structure and the charge storage mechanism of Li-ion half-cell. After full discharge, the liquid alloy is lithiated to form a solid alloy, and its volume expands. During charging, lithium ions are released and the solid alloy gradually returns to the initial liquid state. Figure 9B,D exhibits that the average discharge specific capacity of the eGaIn nanodroplets is 582.6, 532.8, 466.5, 439.1, 402.7, and 379.6 mAh g^−1^ at the current densities of 0.1, 0.2, 0.5, 1.0, 2.0, and 3.0 A g^−1^, respectively, meaning excellent rate performance (65.2% after the current density increased 30 times). As shown in Figure 9C, the discharge specific capacities of these three cycles at 0.1 A g^−1^ are 889, 623.1, and 597.5 mAh g^−1^, respectively. The first cycle shows high discharge specific capacity due to the existence of an irreversible reaction and the formation of an SEI layer. Moreover, the contours of the discharge and charge curves are similar to those previously reported [17,18,19,20], representing the gradual lithiation/delithiation of Ga/In through the intermediate alloy.

The excellent performance of the eGaIn nanodroplets was further verified by comparison with the bulk eGaIn alloy. The eGaIn nanodroplets exhibit at least 80% higher reversible capacity than bulk eGaIn alloy at various current densities (Figure 9E). It is worth noting that the bulk eGaIn alloy has almost no performance at high current density. In addition, Figure 9E shows that the performance of the bulk eGaIn alloy begins to decline rapidly after 20 cycles, and there is almost no performance after 60 cycles. Correspondingly, the performance of the eGaIn nanodroplets starts to decline after 40 cycles and stabilizes at around 151 mAh g^−1^, meaning the eGaIn nanodroplets have better cycle stability than bulk eGaIn liquid. Figure 9F further confirms the excellent cycle stability of the eGaIn nanodroplets. The long-term cycle test shows a stable specific capacity retention of the eGaIn nanodroplets after stabilization of 88.6% (100–600 cycle). Based on the above analyses, the eGaIn nanodroplets exhibit better performance, which can be attributed to the following possible reasons: (1) The nanodroplets increase the exposure area of the electroactive material, and make more materials participate in the reaction. (2) The nanodroplets’ structure not only facilitates easy electrolyte access, but also reduces the ion diffusion transport distance, making the ion diffusion fast within active materials. (3) Bulk eGaIn alloy has a huge volume expansion (about 160%) after full lithiation, causing the bulk material to fall off the current collector. The eGaIn nanodroplets are beneficial to relieve the stress induced by lithiation/delithiation of eGaIn. The carbon black is also beneficial to protect the nanodroplets deposited on the bottom from falling off the current collector.

## 4. Conclusions

The preparation process of liquid eGaIn alloy nanodroplets has been systematically investigated by an ultrasonic method, especially the effects of ultrasonic amplitude, the concentration of surfactant, ultrasonic oscillation time, ultrasonic duration, and rotation speed. In order to obtain droplets with small size, the experimental conditions should be optimized as follows: high amplitude, moderate concentration of surfactant, long ultrasonic time, and short duration of single ultrasonic oscillation. Successive centrifugation of the original solution is required to further achieve high-quality uniform liquid eGaIn alloy nanodroplets with narrow size distribution and small average size below 100 nm. Furthermore, we applied eGaIn nanodroplets as an anode active material for Li-ion batteries, which shows higher specific capacity than bulk eGaIn under various current densities.

## Data Availability

The data presented in this study are available on request from the corresponding author.
